# Trimming of nails in healthy dogs does not change gait parameters when comparing pre- and post-nail trim

**DOI:** 10.3389/fvets.2026.1728382

**Published:** 2026-02-05

**Authors:** Kirsten Häusler, Katja Söhnel

**Affiliations:** 1 Zentrum für Tierphysiotherapie, Stuttgart, Germany; 2 Institut für spezielle Zoologie und Evolutionsforschung, Friedrich-Schiller Universität Jena, Jena, Germany

**Keywords:** animal welfare, canine biomechanics, canine gait analysis, claw trimming, instrumented treadmill, locomotion, nail clipping, retrospective study

## Abstract

Claw length in dogs is widely assumed to influence gait, with the belief that excessively long claws may alter locomotion patterns and lead to musculoskeletal issues. Still today, this hypothesis lacks objective data. This is the first study to investigate the potential impact of claw length on canine gait using validated pressure-sensing treadmill analysis in a small cohort of clinically healthy dogs, both before and after claw trimming. Contrary to common assumptions, no significant differences in gait parameters, such as stride length, stance phase duration, or vertical ground reaction forces, were observed before and after claw trimming. Length of the front paws was 2 mm longer before nail trimming (*p* = 0.022), and the medio-lateral center of pressure movement was slightly decreased by 1% of paw width before nail trimming (*p* = 0.018). The observed absence of differences in temporospatial and kinetic parameters suggests that, although local paw-level mechanics such as medio-lateral COP or paw length may be affected by claw length, these alterations do not translate into functional changes in global gait parameters. This supports the conclusion that, in clinically sound dogs, longer claws do not impair overall locomotor function. These findings suggest that long claws may not be the cause of altered gait, but rather a consequence of decreased natural abrasion due to abnormal or inefficient gait. This insight shifts the focus from claw length as a primary problem to underlying biomechanical issues that may hinder normal claw abrasion. Further studies are needed to explore the relationship between gait quality and claw health.

## 1 Introduction

Trimming a dog’s claws is widely acknowledged as an essential aspect of canine welfare (1). Data from primary veterinary care records in England indicate that overgrown nails are a prevalent concern, ranking third among the most common issues, affecting 7.1% of dogs (2). This is further supported by a larger 2016 study, which found overgrown nails to be the fourth most frequent health problem with a prevalence of 5.5% (3). In a survey of 35 dog owners, 71% considered claw trimming necessary for their pets. Among these, 48% reported trimming their dog’s nails two to five times annually, while 40% did it even more frequently (1).

Despite their importance for animal welfare, little is known about the natural growth and wear patterns of canine claws. A 12-year longitudinal study of 27 beagles aged 1 to 16 years documented nail growth rates, revealing steady or slightly increased growth up to 3 years of age. Following this, a gradual decline was observed, with an annual decrease of approximately 3%. This resulted in a decline in weekly growth from approximately 2.0 mm at age three to around 1.0 mm by age 15 (4).

Multiple factors can lead to excessive nail growth, including limited movement on hard surfaces, confinement or restricted exercise, age-related mobility issues, injuries, or disorders affecting the paws or claws. Breed-specific differences in limb anatomy and gait patterns further influence growth rates and claw maintenance needs (1). Experimental evidence also suggests that individual factors, such as age, general health, and activity levels, significantly affect natural claw abrasion and maintenance requirements.

While nail injuries in dogs are generally uncommon (5), digits and forelimb injuries are more frequently reported in canine athletes involved in sports like agility, flyball, or canicross (6–9) and have been associated with long nails via subjective reporting (9). However, the role of long nails or poor nail health as risk factors for such injuries remains unclear, especially in pet dogs.

Although not scientifically validated, it is generally believed that long nails that contact the ground can alter a dog’s posture, as the dog likely shifts its weight backward to reduce nail pressure. This shift results in more weight bearing on the larger metapodial pad, rather than the digital pads, suggesting a more plantigrade stance. To date, there is no systematic research on how claw trimming affects a dog’s gait, partly because overgrown nails are among the most underfunded issues in canine health (10). This highlights a significant knowledge gap regarding the relationship between claw maintenance and biomechanical function in dogs.

As objective gait analysis is increasingly integrated into clinical and rehabilitation settings, instrumented treadmills provide a standardized and efficient method for evaluating locomotion under controlled conditions and steady velocities (e.g., steady-state analysis; see relevant sources). This method reduces variability, enabling reliable comparisons of biomechanical parameters over time or across different conditions. An instrumented treadmill can extract valuable data from routine clinical practice without requiring additional experimental animal trials. In this way, instrumented treadmill systems enhance both clinical and scientific rigor and animal welfare by adhering to the principles of the 3Rs (Replacement, Reduction, Refinement), reducing the need for invasive or stressful procedures in prospective studies.

A retrospective review of canine patients who underwent both gait analysis and nail trimming could offer new insights into the functional role of claws in movement. By analyzing temporospatial parameters and ground reaction forces before and after nail trimming in a consistent, controlled treadmill environment, such a study may help determine whether claw length directly influences gait mechanics or if other factors, like posture or movement inefficiencies, contribute to claw overgrowth.

We hypothesize that long claws increase overall paw length. Contrary to common assumptions, we further hypothesize that temporospatial gait parameters remain unaffected by overgrown claws. Additionally, we anticipate no significant changes in paw pressure distribution related to claw length.

## 2 Materials and methods

### 2.1 Animals

A group of privately owned dogs of various breeds, sizes, and conformations (see Table 1) was analyzed using an instrumented treadmill to assess gait characteristics before and after claw trimming. All dogs were presented by their owners to the Zentrum für Tierphysiotherapie located in Stuttgart, Germany, a veterinary physiotherapy center that routinely performs nail trimming as part of regular care. The nails were subjectively assessed to be long. Nails were trimmed incrementally using a conventional guillotine-style nail clipper, following the natural curvature of the claw and maintaining a slight distal angle. For dark-colored nails, trimming was performed cautiously in small steps. Each cut was visually inspected for the appearance of a central black dot, indicating proximity to the vascularized quick, to prevent injury. On average, between 5 mm and 8 mm was removed. The same experienced individual trimmed all claws to ensure consistency. The dogs included in the study had no known orthopedic or neurological impairments and underwent gait analysis as part of their routine assessment at the facility. The study included adult dogs (≥12 months) with no known orthopedic or neurological impairments. Inclusion criteria for the retrospective data analysis comprised: (1) claw trimming without injury, (2) availability of gait data from at least 10 gait cycles recorded pre- and post-trimming on the instrumented treadmill, (3) known withers height and body weight for data normalization.

**Table 1:** Characteristics of the dogs included in the study.

Dog No.	Breed	Weight in kg	Height in cm	Age
1	German shepherd	24	61	2.5 y
2	Rottweiler	36	59	5 y
3	Malinois	27.6	61	4 y
4	Flat coated retriever	31	61	4 y
5	Rottweiler–Bavarian mountain hound mix	28	60	6.5 y
6	Pyrenean shepherd	15.6	50	5 y
7	Border collie	15	52	4 y

### 2.2 Data acquisition

Canine gait data were collected using the zebris CanidGait® (zebris Medical GmbH, Isny, Germany) treadmill system, which features an instrumented treadmill with a capacitive pressure-sensor matrix (9,216 sensors) and a sampling rate of 200 Hz, with synchronized video capability. Data acquisition was performed using a pressure-sensitive walkway system with a sensor resolution of 8.458 × 8.458 mm.

As is well known, speed and gait can affect gait parameters (11). The facility routinely collected multiple measurements at different treadmill speeds. Pre- and post-trimming measurements were performed under identical conditions: for every pre-trim speed, a corresponding post-trim speed was recorded, with paired speeds differing by no more than 10%. Treadmill data were recorded for up to 30 s, with at least 10 gait cycles per speed analyzed, and any cycles during which one or more paws left the sensor platform were excluded. This methodological approach has been well documented in previous studies, which report reliable and reproducible measurement of gait parameters using treadmill-based systems in dogs (12–14). All pre- and post-trim recordings for each dog were performed at the same treadmill speed setting. To quantify speed consistency, the coefficient of variation was calculated from the mean and standard deviation for each trial. The maximum observed variability was 7.6%, which is below the commonly accepted threshold of 10%.

The Software Animal Analysis Suite (zebris Medical GmbH, Isny, Germany) automatically computed spatiotemporal and kinetic gait parameters, including stride length, stride frequency, stance and swing duration, vertical ground reaction forces, center of pressure (COP) trajectories, and vertical impulse for each limb and trial. Parameters relevant to the present study were exported for further analysis.

To determine the peak pressure values, an averaged pressure map was generated for each paw by combining data from all recorded gait cycles per trial. Within this mean distribution, the maximum pressure value (“peak pressure”) for each digital pad and metapodial pad was identified.

To ensure comparability across dogs of varying body sizes, all temporal, spatial, and kinetic parameters were normalized according to the dimensionless approach proposed by Hof (15). Withers’ height (SH) was used as a proxy for limb length. Including body mass ensures that forces are expressed relative to each dog’s weight, allowing comparison across individuals. The normalization was performed as follows:

- Normalized velocity (Froude number):

$\mathrm{Fr}=\hat{v}=\frac{v}{\sqrt{g*\mathrm{SH}}}$ where v is the forward velocity (in m/s), g is gravitational acceleration (9.81 m/s^2^), and SH is withers height.

- Normalized temporal parameters (e.g., stride duration, stance duration, swing duration):

$\hat{t}=\frac{t}{\sqrt{\frac{\mathrm{SH}}{g}}}$ where t is the duration in seconds.

- Normalized spatial parameters (e.g., stride length, stance length, swing length):

$\hat{l}=\frac{l}{\mathrm{SH}}$ where l is the measured length in meters.

- Normalized vertical force:

$\hat{F}=\frac{F}{m*g}\;$ where F is the vertical ground reaction force and m is the body mass.

- Normalized impulse:

$\hat{I}=\frac{I}{m*g*\sqrt{\frac{\mathrm{SH}}{g}}},$ where I is vertical impulse (N·s).

COP values are expressed as a percentage of paw length or width (craniocaudal and mediolateral, respectively).

Left and right limb values were averaged for forelimbs and hindlimbs to reduce asymmetry-related noise. All normalized parameters were subsequently used in statistical modeling.

### 2.3 Statistical analysis

Analyses were conducted using R 4.4.2 within the RStudio environment. To assess the impact of claw length on gait parameters, linear mixed-effects models (LMMs) were fitted with the lmer() function from the lme4 package in R (16). Each gait parameter (e.g., vertical impulse, stance time, peak vertical force) was modeled separately as the dependent variable. Fixed effects included claw length (factor) and velocity (Froude). To account for repeated measurements within individual dogs, dog identity (Name) was included as a random intercept.

In addition to the full dataset, a subset dataset was created that included only pre-trim measurements in which digits 3 and 4 of the forepaws were clearly visible in the contact pressure data. These pre-trim measurements were then compared with their speed-corresponding post-trim measurements. The statistical approach, including model structure and evaluation procedures, remained unchanged for this subset analysis.

Model assumptions (normality, homoscedasticity, and influential data points) were visually checked using residual plots and Q–Q plots. The significance of fixed effects was assessed using likelihood ratio tests and confidence interval analyses. *p*-values were computed using the lmerTest package.

### 2.4 Ethical approval statement

Ethical approval was not required for the studies involving animals, in accordance with local legislation and institutional requirements, because this study involved a retrospective analysis of non-invasive routine clinical data collected during standard veterinary care. According to § 7 of the German Animal Welfare Act (Tierschutzgesetz), retrospective analyses using anonymized data do not constitute animal experiments and therefore do not require approval by an animal ethics committee. No additional invasive interventions or procedures were performed for research purposes. Written informed consent was obtained from all owners for the anonymized use of their animals’ clinical data. Written informed consent was obtained from the owners for their animals’ participation in this study.

## 3 Results

Seven dogs (six female, one male) mean of various breeds took part in the study. The dogs varied in body size, weight, and age, with a mean height at the withers of 57.8 ± 5.7 cm and an average body weight of 25.3 ± 7.7 kg. Their ages ranged from 2.5 to 6.5 years. Breeds included the German Shepherd, Rottweiler, Malinois, Flat-Coated Retriever, Border Collie, Pyrenean Shepherd, and a Rottweiler–Bavarian Mountain Hound mix (see Table 1). A total of 137 trials were analyzed; however, only one dog performed the walking task. In contrast, all the other dogs walked and trotted on the instrumented treadmill at various speeds, except for one dog that completed only one walking and one trotting trial before and after claw trimming.

**Figure 1:**
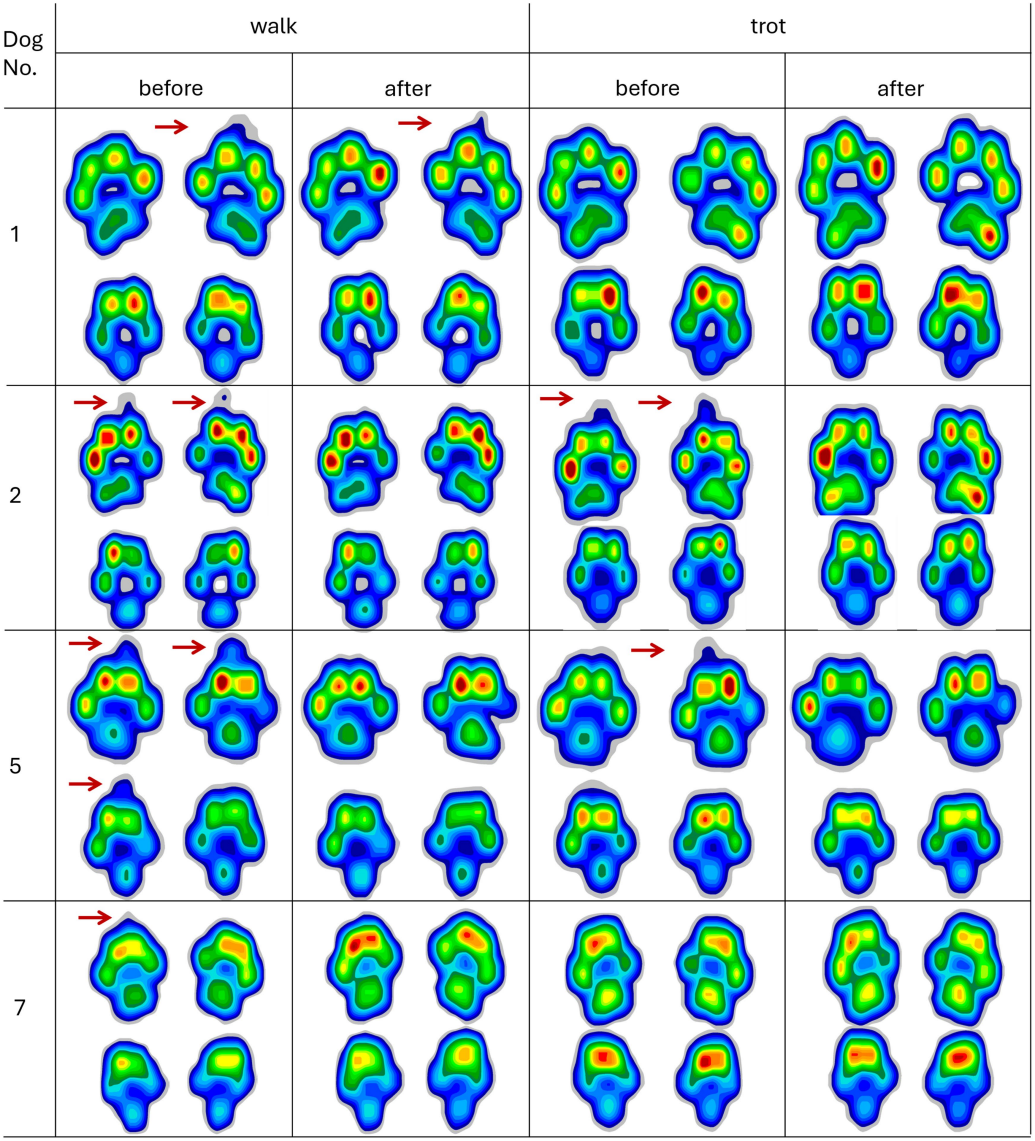
Paw contact pressure of four dogs with visible claws detected during walking (left) and trotting (right) before and after claw trimming. Red arrows point to the claws’ contact with the pressure mat. Colors from blue to red indicate increasing pressure.

All the dogs had at least one claw in contact with the floor while standing. In every dog, the medial front claw (2nd digit) touched the ground. In four of them, the front claws, three and/or four, also contacted the ground during walking. Of those four dogs, two showed claw contact in all measurements across all speeds, and the other two showed claw contact only during walking. Those four dogs were included in the subset (see Figure 1, red arrows).

Before claw trimming, forepaw length was on average approximately 2.2 mm greater than after trimming, indicating a small but statistically significant effect of claw status (*p* = 0.0229), while no statistical effect was seen in the hindlimbs. Contrary to the width of the hind paws, which was 1 mm smaller before claw trimming (*p* = 0.018), there was no statistically significant effect in the forepaws (see Table 2). From the subset of the measurements with visible claw contact (33 trials pre and post trimming, respectively), again a significantly longer pawprint was measured on the forelimbs before claw trimming, with an estimated 6 mm longer paws prior to trimming (*p* < 0.001). Therefore, the paw contact area was 2 cm^2^ greater before claw trimming (*p* = 0.024) (see Supplementary file S2).

**Table 2:** Results of the mixed effect models for the parameters of the forepaws, from seven dogs.

Variable	Limb	Term	Estimate	Std. error	*t* value	*p* value	Conf. low	Conf. high	*R*^2^ marginal	*R*^2^ Cond.	Variance dog	Variance residual
Relative stride duration	Front	Intercept	1.972	0.035	57.141	0.000	1.901	2.043	0.830	0.888	0.004	0.007
		Froude	−0.929	0.031	−30.329	0.000	−0.990	−0.869				
		ClawBefore	−0.001	0.015	−0.059	0.953	−0.030	0.028				
Relative stance duration	Front	Intercept	1.394	0.026	54.615	0.000	1.342	1.446	0.889	0.917	0.002	0.005
		Froude	−0.905	0.025	−36.597	0.000	−0.954	−0.856				
		ClawBefore	−0.005	0.012	−0.392	0.696	−0.028	0.019				
Relative swing duration	Front	Intercept	0.577	0.015	39.382	0.000	0.546	0.608	0.022	0.486	0.001	0.001
		Froude	−0.025	0.011	−2.176	0.031	−0.047	−0.002				
		ClawBefore	0.004	0.005	0.713	0.477	−0.007	0.014				
Relative stride length	Front	Intercept	0.451	0.013	33.470	0.000	0.422	0.480	0.936	0.976	0.001	0.001
		Froude	0.590	0.008	69.483	0.000	0.573	0.607				
		ClawBefore	−0.006	0.004	−1.617	0.108	−0.014	0.001				
Relative peak vertical force	Front	Intercept	28.354	3.145	9.017	0.000	21.720	34.988	0.843	0.910	36.879	49.990
		Froude	87.271	2.564	34.035	0.000	82.199	92.343				
		ClawBefore	−0.035	1.216	−0.029	0.977	−2.441	2.371				
Paw contact area	Front	Intercept	50.220	4.119	12.191	0.000	40.700	59.740	0.183	0.922	111.056	11.734
		Froude	21.187	1.251	16.937	0.000	18.712	23.662				
		ClawBefore	0.441	0.590	0.748	0.456	−0.726	1.607				
Paw length	Front	Intercept	93.532	4.855	19.265	0.000	82.512	104.55	0.149	0.851	144.785	30.753
		Froude	22.129	2.024	10.934	0.000	18.125	26.132				
		ClawBefore	2.209	0.954	2.314	0.022	0.320	4.097				
Paw width	Front	Intercept	71.704	3.024	23.713	0.000	64.735	78.674	0.021	0.897	59.401	6.993
		Froude	4.793	0.966	4.963	0.000	2.882	6.703				
		ClawBefore	−0.298	0.455	−0.654	0.514	−1.198	0.603				
Relative COP path length	Front	Intercept	6.352	0.332	19.156	0.000	5.623	7.081	0.015	0.685	0.588	0.277
		Froude	−0.397	0.192	−2.073	0.040	−0.777	−0.018				
		ClawBefore	−0.122	0.091	−1.343	0.182	−0.301	0.058				
Relative cranio-caudal COP path	Front	Intercept	31.232	1.719	18.166	0.000	27.678	34.786	0.012	0.312	8.385	19.212
		Froude	1.968	1.583	1.243	0.216	−1.164	5.099				
		ClawBefore	−0.548	0.754	−0.727	0.468	−2.039	0.943				
Relative medio-laterl COP path	Front	Intercept	16.363	1.731	9.451	0.000	12.442	20.283	0.080	0.833	18.314	4.059
		Froude	−5.427	0.735	−7.382	0.000	−6.881	−3.973				
		ClawBefore	−0.830	0.347	−2.395	0.018	−1.516	−0.144				

**Figure 2:**
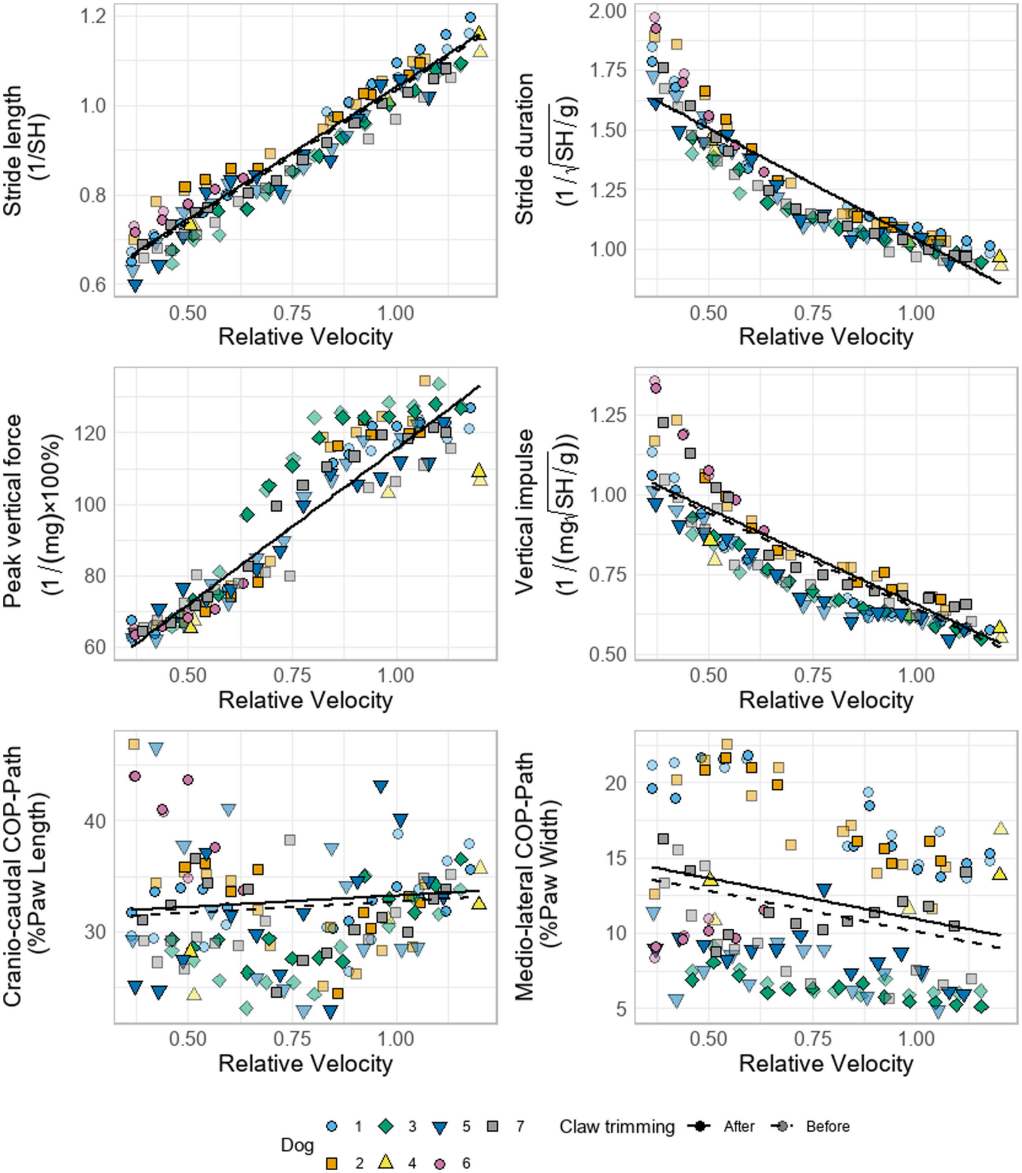
Relationship between gait parameters and relative velocity (Froude number) in seven dogs before and after claw trimming. Data points represent individual measurements for each dog, with different shapes denoting individual dogs. The strong (darker) colors represent the claw status after trimming, while lighter shades indicate the status before trimming. Lines depict predicted gait parameters from the linear mixed-effects model for each claw condition: before (dashed) and after (solid) trimming, illustrating the effect of claw trimming on gait dynamics while controlling for speed variation. No significant effects of claw trimming were detected, except medio-lateral COP path.

All tested temporospatial parameters showed no significant differences before and after claw trimming. Peak vertical force and impulse did not differ significantly in either the hind limbs or the forelimbs (Figure 2). There was no statistically significant effect of claw trimming the fore-hind weight distribution. For the COP parameters, only the mediolateral displacement of the COP in the forelimbs was <1% lower before nail trimming in both datasets. Including the full dataset confirms the previously observed reduction in medio-lateral COP excursion following claw trimming. While statistical significance increases from *p* = 0.039 to *p* = 0.018 with the larger sample, the effect size remains small, and the majority of variance is still explained by inter-individual differences between dogs, see Supplementary files S1 and S2.

**Figure 3:**
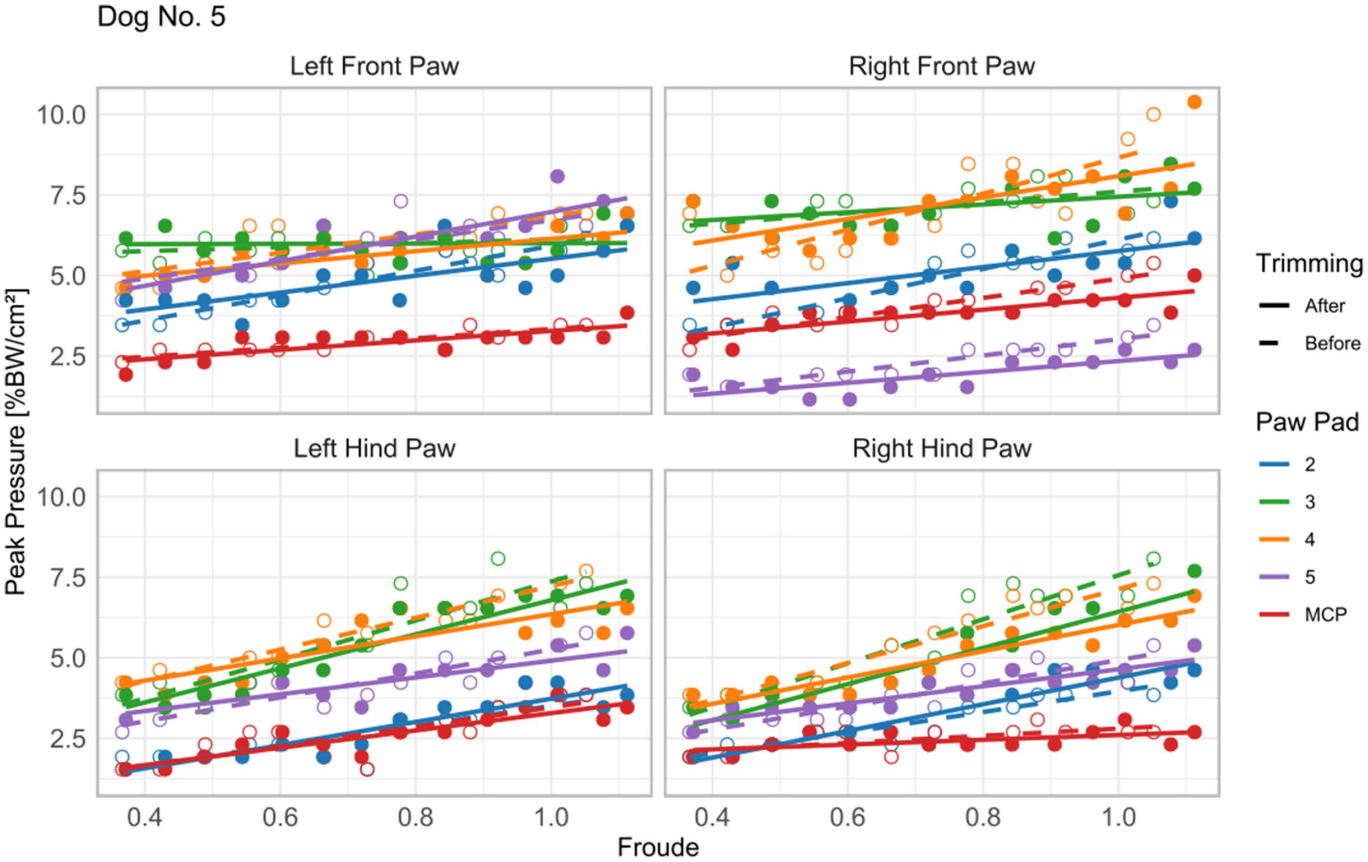
Peak pressure under the pads of Dog No. 5 in relation to the relative velocity Froude. Dog No. 5 showed visible claw contact in the pressure map across all speeds. Open circles represent peak pressure under the pad before trimming, while closed circles represent the pressure after trimming. Dashed and solid lines show the linear fit before and after claw trimming, respectively. Blue: second digital pad; Green: third digital pad; Yellow: fourth digital pad; Violet: fifth digital pad; Red: metapodial pad (MP).

Froude number showed highly significant effects (*p* < 0.001) on all the gait parameters except for swing duration and craniocaudal COP path, see Figure 3, Table 2 and Supplementary file S1 and S2.

## 4 Discussion

This retrospective study is the first to systematically assess the impact of trimming long claws on biomechanical gait parameters in dogs. We analyzed 137 locomotion trials, each comprising at least 10 gait cycles, from seven dogs (68 trials before and 69 after claw trimming). All trials were conducted immediately before and after claw trimming on an instrumented treadmill, with speeds ranging from 2.9 to 10.1 km/h. In line with the dynamic similarity hypothesis, all gait parameters were normalized by body weight and limb length (withers height), ensuring comparability across subjects and speeds.

All dogs exhibited at least one long claw that was in contact with the ground during standing. Consistently, the front claws are longer than the rear ones across all subjects. This disparity may be attributed to the biomechanics of locomotion, in which the accelerating action of the hind limbs generates greater propulsive forces, increasing pressure on the hind paws during acceleration or sprint initiation (17). Furthermore, as observed in cats, the growth rate of the rear claws is slower compared to the front claws (18), a pattern that might similarly apply to dogs.

Four of the seven dogs exhibited visible claw contact in the paw pressure distributions during walking, with two also showing claw contact during trotting (see Figure 1). Consistent with our initial hypothesis, statistical analysis revealed a significantly longer paw length before trimming. One possible explanation for the absence of visible claw contact in some dogs is that they alter their stance-phase posture, shifting weight from the digital pads to the metapodial pads, thereby reducing direct claw-ground contact. However, this explanation is unlikely, since dogs typically employ a “heel-first” ground-contact pattern, followed by a toe-off, as indicated by the cranial progression of the center of pressure (COP) (19–21). Uncomfortable claw contact is therefore more plausible at the end of the stance phase, when the third and fourth digital pads peak in force (22). Such a weight shift towards the metapodial pads during late stance would reduce the craniocaudal component of the COP. However, neither parameter changed, and maximum pressure throughout the gait cycle remained unchanged before and after trimming, with consistently lower pressures under the metapodial pads compared to digital pads 3 and 4 (see Figures 1 and 3).

When the full dataset was analyzed, a very similar reduction in medio-lateral COP excursion of approximately 0.83% of paw width was observed. Restricting the analysis to dogs exhibiting visible claw–ground contact yielded a slightly larger reduction of about 1% of paw width prior to claw trimming. Although these differences were statistically detectable, their absolute magnitude was small and should therefore be interpreted cautiously with respect to functional relevance. Notably, while statistical significance increased with larger sample sizes, effect sizes remained consistently small, and inter-individual differences among dogs continued to account for a substantial proportion of the observed variance.

Consistent with our second hypothesis, no statistically significant differences were observed in any measured stride or stance parameter following claw trimming. All mixed-effects models indicated that a large proportion of the total variance was attributable to differences between dogs, as reflected in the intraclass correlation coefficients (ICCs> 0.75) calculated from the variance components. This finding supports the notion that individual locomotor phenotypes are robust and largely unaffected by claw length. Specifically, stride length and stride duration showed no significant changes before versus after trimming (Figure 2). The observed absence of differences in stance and stride durations therefore suggests that, although local paw-level mechanics such as medio-lateral COP or paw length may be affected by claw length, these alterations do not translate into functional changes in global gait parameters. This supports the conclusion that, in clinically sound dogs, longer claws do not impair overall locomotor function.

Peak vertical forces and their distribution between the fore- and hind limbs remained unchanged after claw trimming. This finding is consistent with studies in cattle, where claw trimming did not affect stance, swing, or stride duration, peak vertical force, or the overall progression of ground reaction forces during treadmill locomotion, despite differences in foot morphology (23).

Our results suggest no immediate changes in gait parameters following claw trimming, though several factors could explain this. Firstly, the claws may not have been long enough to induce noticeable postural or gait alterations, as indicated by the mostly adequate physical care ratings on the Tufts Animal Care and Condition Scale. Secondly, any effects on posture and gait might take longer to manifest, as observed in sows 48 h after trimming. (24). Thirdly, claw length might be a consequence rather than a cause of altered locomotion patterns. Dogs with longer claws may exhibit altered gait patterns that reduce contact between the claws and the ground, leading to less abrasion and, consequently, overgrown nails.

Long nails may thus serve as indicators of underlying postural changes or locomotor deficits. For example, corn-like lesions frequently develop on digital pads, causing discomfort and a compensatory shift of weight to the metacarpal pads, which can lead to excessive nail growth (25). Injuries to the digital flexor tendons similarly reduce claw wear, resulting in longer nails (25). Nutritional deficiencies during growth periods have also been linked to alterations in paw structure, such as splayed toes and plantigrade posture, which correlate with increased claw length due to reduced natural wear (26). Conversely, certain neurological disorders that impair proprioception may cause abnormally short nails (25).

Breed-specific differences in nail growth and wear have been documented. Some breeds, including the Chihuahua, Beagle, Greyhound, Pug, and Whippet, have a higher risk of overgrown claws (27–31). Studies of peak vertical forces reveal that German Shepherds, Pit Bulls, Labrador Retrievers, Anatolian Shepherds, and mixed breeds exert higher forces under metacarpal pads. In contrast, Greyhounds exhibit the highest forces under the third and fourth digital pads (22, 32–37). The consistently lowest forces measured under the second digital pad may explain the frequent lengthening of claws at this digit due to reduced abrasion.

Age and activity level also influence claw length. Increased carpal joint extension with advancing age (38) may elevate metacarpal pad loading, thereby reducing natural toe wear. Smaller breeds tend to exercise less frequently and for shorter durations than larger breeds, potentially leading to longer nails (39). Additionally, some hunting breeds kept as pets may be more prone to overgrown nails due to reduced activity relative to their original working purpose. Greyhounds, bred initially for racing but now increasingly kept as pets in the UK, often exhibit this nail issue, possibly linked to orthopedic injuries and decreased exercise on hard surfaces later in life (28).

Finally, our data underscored a strong effect of relative velocity on all gait parameters (Figures 2 and 3, Table 2 and Supplementary files S1 and S2), consistent with previous canine biomechanical studies. Increased velocity shortens stance phase duration and lengthens stride length, while swing phase duration remains largely speed-independent (11, 40). Peak vertical force and impulse, which correlate with stance time, also vary with locomotor speed. Consequently, controlling for velocity is essential when analyzing gait to ensure that observed changes reflect experimental effects, such as claw trimming, rather than confounding speed differences.

A key limitation of this retrospective study is the absence of a standardized protocol. Nevertheless, consistent workflows were followed by the same animal physiotherapist, resulting in a high-quality dataset and robust findings using linear mixed models (lmer). Given the repeated-measures pre–post design and the high ICC values observed, linear mixed-effects models with dog as a random effect were considered appropriate.

The sample size was limited to seven dogs, which restricts generalizability and the power to detect subtle effects. Although gait parameters were averaged over at least 10 gait cycles per limb pair, individual anatomical and locomotor variability may still have influenced the results. The retrospective nature of the study inherently limits experimental control. While treadmill speeds were predefined and the treadmill was calibrated, complete standardization was unattainable. Limited gait records in one may have introduced minor data quality issues. Averaging gait parameters reduces stride-to-stride variability but might mask transient effects. The small sample size and the limited number of trials in some dogs could increase the risk of Type II errors, leading to the failure to detect small but potentially meaningful effects.

In addition, only the immediate effects of claw trimming were assessed. Potential delayed adaptations in posture or gait related to the viscoelastic properties of soft tissues could not be evaluated, as no follow-up measurements were available. Consequently, changes that may emerge hours or days after trimming cannot be excluded and should be addressed in future prospective studies.

Furthermore, claw length was not objectively quantified before or after trimming, nor was the proportion of claw removed measured. These data were not routinely documented in the clinical records and therefore could not be reconstructed retrospectively. This represents a major limitation of the study and limits the ability to relate the magnitude of claw trimming to biomechanical outcomes. Objective measurements of claw length and standardized trimming protocols should be incorporated in future prospective investigations.

## Data Availability

The raw data supporting the conclusions of this article will be made available by the authors, without undue reservation.
