# Supplementary material for: Trimming of nails in healthy dogs does not change gait parameters when comparing pre- and post-nail trim
Source: Front Vet Sci. 2026 Feb 5;13:1728382. doi: 10.3389/fvets.2026.1728382 (PMC12916395; doi:10.3389/fvets.2026.1728382)
Supplement: Supplementary file 1 [file Table_1.docx]

## Supplementary Tables

Table S1 containing results from the statistical model applied on the whole dataset

Table S2 containing results from the subset. only four dogs included that showed a visible claw contact on the pressure mat. 33 measurements before nail trimming and 33 measurements after nail trimming at comparable speeds

## S1 Statistical result from linear mixed effect model of the full dataset

| **Variable** | **limb** | **Term** | **Estimate** | **Std. Error** | **t value** | **p value** | **Conf. Low** | **Conf. High** | **R^2^ Marginal** | **R^2^ Cond.** | **Variance Dog** | **Variance Residual** |
| --- | --- | --- | --- | --- | --- | --- | --- | --- | --- | --- | --- | --- |
| relative stride duration | front | Intercept | 1.972 | 0.035 | 57.141 | 0.000 | 1.901 | 2.043 | 0.830 | 0.888 | 0.004 | 0.007 |
|  |  | Froude | -0.929 | 0.031 | -30.329 | 0.000 | -0.990 | -0.869 |  |  |  |  |
|  |  | ClawBefore | -0.001 | 0.015 | -0.059 | 0.953 | -0.030 | 0.028 |  |  |  |  |
|  | hind | Intercept | 1.976 | 0.035 | 57.093 | 0.000 | 1.905 | 2.047 | 0.829 | 0.886 | 0.004 | 0.007 |
|  |  | Froude | -0.934 | 0.031 | -30.091 | 0.000 | -0.995 | -0.872 |  |  |  |  |
|  |  | ClawBefore | -0.001 | 0.015 | -0.070 | 0.945 | -0.030 | 0.028 |  |  |  |  |
| relative stance duration | front | Intercept | 1.394 | 0.026 | 54.615 | 0.000 | 1.342 | 1.446 | 0.889 | 0.917 | 0.002 | 0.005 |
|  |  | Froude | -0.905 | 0.025 | -36.597 | 0.000 | -0.954 | -0.856 |  |  |  |  |
|  |  | ClawBefore | -0.005 | 0.012 | -0.392 | 0.696 | -0.028 | 0.019 |  |  |  |  |
|  | hind | Intercept | 1.355 | 0.029 | 46.104 | 0.000 | 1.294 | 1.415 | 0.871 | 0.910 | 0.002 | 0.006 |
|  |  | Froude | -0.939 | 0.027 | -34.767 | 0.000 | -0.993 | -0.886 |  |  |  |  |
|  |  | ClawBefore | 0.004 | 0.013 | 0.337 | 0.736 | -0.021 | 0.030 |  |  |  |  |
| relative swing duration | front | Intercept | 0.577 | 0.015 | 39.382 | 0.000 | 0.546 | 0.608 | 0.022 | 0.486 | 0.001 | 0.001 |
|  |  | Froude | -0.025 | 0.011 | -2.176 | 0.031 | -0.047 | -0.002 |  |  |  |  |
|  |  | ClawBefore | 0.004 | 0.005 | 0.713 | 0.477 | -0.007 | 0.014 |  |  |  |  |
|  | hind | Intercept | 0.621 | 0.015 | 40.660 | 0.000 | 0.589 | 0.653 | 0.004 | 0.333 | 0.001 | 0.001 |
|  |  | Froude | 0.005 | 0.014 | 0.398 | 0.691 | -0.022 | 0.033 |  |  |  |  |
|  |  | ClawBefore | -0.005 | 0.007 | -0.823 | 0.412 | -0.018 | 0.008 |  |  |  |  |
| relative stride length | front | Intercept | 0.451 | 0.013 | 33.470 | 0.000 | 0.422 | 0.480 | 0.936 | 0.976 | 0.001 | 0.001 |
|  |  | Froude | 0.590 | 0.008 | 69.483 | 0.000 | 0.573 | 0.607 |  |  |  |  |
|  |  | ClawBefore | -0.006 | 0.004 | -1.617 | 0.108 | -0.014 | 0.001 |  |  |  |  |
|  | hind | Intercept | 0.452 | 0.013 | 33.670 | 0.000 | 0.423 | 0.481 | 0.936 | 0.976 | 0.001 | 0.001 |
|  |  | Froude | 0.589 | 0.008 | 69.410 | 0.000 | 0.572 | 0.606 |  |  |  |  |
|  |  | ClawBefore | -0.007 | 0.004 | -1.636 | 0.104 | -0.014 | 0.001 |  |  |  |  |
| relative peak vertical force | front | Intercept | 28.354 | 3.145 | 9.017 | 0.000 | 21.720 | 34.988 | 0.843 | 0.910 | 36.879 | 49.990 |
|  |  | Froude | 87.271 | 2.564 | 34.035 | 0.000 | 82.199 | 92.343 |  |  |  |  |
|  |  | ClawBefore | -0.035 | 1.216 | -0.029 | 0.977 | -2.441 | 2.371 |  |  |  |  |
|  | hind | Intercept | 23.900 | 2.383 | 10.031 | 0.000 | 18.921 | 28.879 | 0.774 | 0.870 | 21.180 | 28.687 |
|  |  | Froude | 52.783 | 1.942 | 27.173 | 0.000 | 48.941 | 56.625 |  |  |  |  |
|  |  | ClawBefore | -0.112 | 0.921 | -0.121 | 0.904 | -1.934 | 1.711 |  |  |  |  |
| relative vertical impulse | front | Intercept | 1.256 | 0.035 | 36.319 | 0.000 | 1.180 | 1.331 | 0.695 | 0.879 | 0.006 | 0.004 |
|  |  | Froude | -0.601 | 0.023 | -26.607 | 0.000 | -0.645 | -0.556 |  |  |  |  |
|  |  | ClawBefore | -0.013 | 0.011 | -1.216 | 0.226 | -0.034 | 0.008 |  |  |  |  |
|  | hind | Intercept | 0.774 | 0.020 | 38.603 | 0.000 | 0.730 | 0.817 | 0.747 | 0.895 | 0.002 | 0.001 |
|  |  | Froude | -0.396 | 0.013 | -29.545 | 0.000 | -0.423 | -0.370 |  |  |  |  |
|  |  | ClawBefore | 0.004 | 0.006 | 0.643 | 0.521 | -0.008 | 0.017 |  |  |  |  |
| paw contact area | front | Intercept | 50.220 | 4.119 | 12.191 | 0.000 | 40.700 | 59.740 | 0.183 | 0.922 | 111.056 | 11.734 |
|  |  | Froude | 21.187 | 1.251 | 16.937 | 0.000 | 18.712 | 23.662 |  |  |  |  |
|  |  | ClawBefore | 0.441 | 0.590 | 0.748 | 0.456 | -0.726 | 1.607 |  |  |  |  |
|  | hind | Intercept | 44.418 | 3.227 | 13.764 | 0.000 | 36.986 | 51.849 | 0.095 | 0.897 | 67.223 | 8.627 |
|  |  | Froude | 11.326 | 1.072 | 10.561 | 0.000 | 9.204 | 13.448 |  |  |  |  |
|  |  | ClawBefore | -0.508 | 0.506 | -1.005 | 0.317 | -1.508 | 0.492 |  |  |  |  |
| paw length | front | Intercept | 93.532 | 4.855 | 19.265 | 0.000 | 82.512 | 104.55 | 0.149 | 0.851 | 144.785 | 30.753 |
|  |  | Froude | 22.129 | 2.024 | 10.934 | 0.000 | 18.125 | 26.132 |  |  |  |  |
|  |  | ClawBefore | 2.209 | 0.954 | 2.314 | 0.022 | 0.320 | 4.097 |  |  |  |  |
|  | hind | Intercept | 91.393 | 3.700 | 24.698 | 0.000 | 83.198 | 99.589 | 0.129 | 0.746 | 75.462 | 31.123 |
|  |  | Froude | 16.063 | 2.033 | 7.900 | 0.000 | 12.041 | 20.086 |  |  |  |  |
|  |  | ClawBefore | 0.021 | 0.960 | 0.021 | 0.983 | -1.879 | 1.920 |  |  |  |  |
| paw width | front | Intercept | 71.704 | 3.024 | 23.713 | 0.000 | 64.735 | 78.674 | 0.021 | 0.897 | 59.401 | 6.993 |
|  |  | Froude | 4.793 | 0.966 | 4.963 | 0.000 | 2.882 | 6.703 |  |  |  |  |
|  |  | ClawBefore | -0.298 | 0.455 | -0.654 | 0.514 | -1.198 | 0.603 |  |  |  |  |
|  | hind | Intercept | 59.940 | 2.464 | 24.323 | 0.000 | 54.275 | 65.605 | 0.047 | 0.882 | 38.899 | 5.487 |
|  |  | Froude | 5.574 | 0.855 | 6.517 | 0.000 | 3.882 | 7.266 |  |  |  |  |
|  |  | ClawBefore | -0.969 | 0.403 | -2.403 | 0.018 | -1.767 | -0.171 |  |  |  |  |
| relative COP path length | front | Intercept | 6.352 | 0.332 | 19.156 | 0.000 | 5.623 | 7.081 | 0.015 | 0.685 | 0.588 | 0.277 |
|  |  | Froude | -0.397 | 0.192 | -2.073 | 0.040 | -0.777 | -0.018 |  |  |  |  |
|  |  | ClawBefore | -0.122 | 0.091 | -1.343 | 0.182 | -0.301 | 0.058 |  |  |  |  |
|  | hind | Intercept | 6.061 | 0.337 | 17.984 | 0.000 | 5.311 | 6.810 | 0.111 | 0.768 | 0.645 | 0.228 |
|  |  | Froude | -1.309 | 0.174 | -7.511 | 0.000 | -1.653 | -0.964 |  |  |  |  |
|  |  | ClawBefore | -0.157 | 0.082 | -1.913 | 0.058 | -0.320 | 0.005 |  |  |  |  |
| relative cranio-caudal COP Path | front | Intercept | 31.232 | 1.719 | 18.166 | 0.000 | 27.678 | 34.786 | 0.012 | 0.312 | 8.385 | 19.212 |
|  |  | Froude | 1.968 | 1.583 | 1.243 | 0.216 | -1.164 | 5.099 |  |  |  |  |
|  |  | ClawBefore | -0.548 | 0.754 | -0.727 | 0.468 | -2.039 | 0.943 |  |  |  |  |
|  | hind | Intercept | 28.974 | 2.490 | 11.637 | 0.000 | 23.359 | 34.589 | 0.004 | 0.805 | 37.402 | 9.119 |
|  |  | Froude | 1.202 | 1.102 | 1.091 | 0.277 | -0.977 | 3.382 |  |  |  |  |
|  |  | ClawBefore | -0.627 | 0.520 | -1.206 | 0.230 | -1.655 | 0.401 |  |  |  |  |
| relative medio-laterl COP Path | front | Intercept | 16.363 | 1.731 | 9.451 | 0.000 | 12.442 | 20.283 | 0.080 | 0.833 | 18.314 | 4.059 |
|  |  | Froude | -5.427 | 0.735 | -7.382 | 0.000 | -6.881 | -3.973 |  |  |  |  |
|  |  | ClawBefore | -0.830 | 0.347 | -2.395 | 0.018 | -1.516 | -0.144 |  |  |  |  |
|  | hind | Intercept | 7.981 | 0.838 | 9.524 | 0.000 | 6.170 | 9.793 | 0.128 | 0.660 | 3.470 | 2.215 |
|  |  | Froude | 3.666 | 0.542 | 6.766 | 0.000 | 2.594 | 4.738 |  |  |  |  |
|  |  | ClawBefore | -0.149 | 0.256 | -0.583 | 0.561 | -0.656 | 0.357 |  |  |  |  |
| COP speed | front | Intercept | 0.709 | 0.949 | 0.748 | 0.470 | -1.376 | 2.794 | 0.852 | 0.951 | 4.770 | 2.335 |
|  |  | Froude | 25.734 | 0.557 | 46.222 | 0.000 | 24.633 | 26.835 |  |  |  |  |
|  |  | ClawBefore | -0.235 | 0.263 | -0.894 | 0.373 | -0.755 | 0.285 |  |  |  |  |
|  | hind | Intercept | 1.405 | 0.846 | 1.660 | 0.121 | -0.422 | 3.232 | 0.849 | 0.939 | 3.482 | 2.347 |
|  |  | Froude | 23.043 | 0.558 | 41.332 | 0.000 | 21.940 | 24.146 |  |  |  |  |
|  |  | ClawBefore | -0.451 | 0.264 | -1.712 | 0.089 | -0.973 | 0.070 |  |  |  |  |
| Weight distribution | front/ hind | Intercept | 1.366 | 0.052 | 26.283 | 0.000 | 1.255 | 1.477 | 0.051 | 0.581 | 0.012 | 0.010 |
|  |  | Froude | 0.139 | 0.036 | 3.854 | 0.000 | 0.068 | 0.211 |  |  |  |  |
|  |  | ClawBefore | 0.007 | 0.017 | 0.432 | 0.666 | -0.026 | 0.041 |  |  |  |  |

## S2 Statistical result from linear mixed effect model of the reduced dataset

| **Variable** | **limb** | **Term** | **Estimate** | **Std. Error** | **t value** | **p value** | **Conf. Low** | **Conf. High** | **R^2^ Marginal** | **R^2^ Cond.** | **Variance Dog** | **Variance Residual** |
| --- | --- | --- | --- | --- | --- | --- | --- | --- | --- | --- | --- | --- |
| relative stride duration | front | Intercept | 2.050 | 0.039 | 52.630 | 0.000 | 1.971 | 2.129 | 0.879 | 0.900 | 0.001 | 0.006 |
|  |  | Froude | -1.068 | 0.050 | -21.329 | 0.000 | -1.168 | -0.968 |  |  |  |  |
|  |  | ClawBefore | -0.004 | 0.019 | -0.192 | 0.849 | -0.042 | 0.035 |  |  |  |  |
|  | hind | Intercept | 2.057 | 0.039 | 52.386 | 0.000 | 1.977 | 2.137 | 0.878 | 0.897 | 0.001 | 0.006 |
|  |  | Froude | -1.078 | 0.051 | -21.164 | 0.000 | -1.179 | -0.976 |  |  |  |  |
|  |  | ClawBefore | -0.004 | 0.019 | -0.201 | 0.842 | -0.043 | 0.035 |  |  |  |  |
| relative stance duration | front | Intercept | 1.469 | 0.026 | 56.189 | 0.000 | 1.416 | 1.522 | 0.934 | 0.942 | 0.000 | 0.003 |
|  |  | Froude | -1.017 | 0.035 | -29.374 | 0.000 | -1.086 | -0.947 |  |  |  |  |
|  |  | ClawBefore | -0.016 | 0.013 | -1.197 | 0.236 | -0.043 | 0.011 |  |  |  |  |
|  | hind | Intercept | 1.468 | 0.032 | 46.364 | 0.000 | 1.402 | 1.534 | 0.925 | 0.947 | 0.001 | 0.003 |
|  |  | Froude | -1.112 | 0.038 | -29.479 | 0.000 | -1.187 | -1.036 |  |  |  |  |
|  |  | ClawBefore | -0.003 | 0.014 | -0.245 | 0.807 | -0.032 | 0.025 |  |  |  |  |
| relative swing duration | front | Intercept | 0.579 | 0.016 | 35.322 | 0.000 | 0.545 | 0.613 | 0.106 | 0.329 | 0.000 | 0.001 |
|  |  | Froude | -0.049 | 0.020 | -2.421 | 0.018 | -0.089 | -0.008 |  |  |  |  |
|  |  | ClawBefore | 0.012 | 0.008 | 1.643 | 0.105 | -0.003 | 0.027 |  |  |  |  |
|  | hind | Intercept | 0.593 | 0.016 | 36.540 | 0.000 | 0.560 | 0.626 | 0.024 | 0.032 | 0.000 | 0.001 |
|  |  | Froude | 0.028 | 0.022 | 1.253 | 0.217 | -0.017 | 0.073 |  |  |  |  |
|  |  | ClawBefore | -0.000 | 0.009 | -0.049 | 0.961 | -0.019 | 0.018 |  |  |  |  |
| relative stride length | front | Intercept | 0.469 | 0.017 | 27.733 | 0.000 | 0.432 | 0.506 | 0.920 | 0.958 | 0.001 | 0.001 |
|  |  | Froude | 0.559 | 0.017 | 32.671 | 0.000 | 0.525 | 0.593 |  |  |  |  |
|  |  | ClawBefore | -0.002 | 0.006 | -0.378 | 0.707 | -0.015 | 0.010 |  |  |  |  |
|  | hind | Intercept | 0.472 | 0.017 | 28.255 | 0.000 | 0.435 | 0.508 | 0.921 | 0.959 | 0.001 | 0.001 |
|  |  | Froude | 0.556 | 0.017 | 32.871 | 0.000 | 0.522 | 0.589 |  |  |  |  |
|  |  | ClawBefore | -0.003 | 0.006 | -0.419 | 0.677 | -0.015 | 0.010 |  |  |  |  |
| relative peak vertical force | front | Intercept | 23.379 | 2.278 | 10.262 | 0.000 | 18.831 | 27.928 | 0.933 | 0.933 | 0.000 | 29.369 |
|  |  | Froude | 94.079 | 3.126 | 30.091 | 0.000 | 87.837 | 100.32 |  |  |  |  |
|  |  | ClawBefore | 1.347 | 1.334 | 1.009 | 0.316 | -1.317 | 4.010 |  |  |  |  |
|  | hind | Intercept | 16.076 | 3.261 | 4.930 | 0.001 | 8.713 | 23.439 | 0.811 | 0.922 | 26.753 | 18.830 |
|  |  | Froude | 64.949 | 2.910 | 22.323 | 0.000 | 59.137 | 70.762 |  |  |  |  |
|  |  | ClawBefore | 0.174 | 1.068 | 0.163 | 0.871 | -1.961 | 2.310 |  |  |  |  |
| relative vertical impulse | front | Intercept | 1.294 | 0.036 | 36.375 | 0.000 | 1.220 | 1.368 | 0.786 | 0.852 | 0.002 | 0.004 |
|  |  | Froude | -0.675 | 0.042 | -16.219 | 0.000 | -0.758 | -0.592 |  |  |  |  |
|  |  | ClawBefore | -0.023 | 0.016 | -1.508 | 0.137 | -0.054 | 0.008 |  |  |  |  |
|  | hind | Intercept | 0.781 | 0.016 | 48.815 | 0.000 | 0.749 | 0.814 | 0.865 | 0.883 | 0.000 | 0.001 |
|  |  | Froude | -0.417 | 0.021 | -19.815 | 0.000 | -0.459 | -0.375 |  |  |  |  |
|  |  | ClawBefore | 0.003 | 0.008 | 0.428 | 0.670 | -0.013 | 0.020 |  |  |  |  |
| paw contact area | front | Intercept | 52.529 | 5.428 | 9.677 | 0.000 | 38.306 | 66.752 | 0.202 | 0.920 | 107.707 | 12.029 |
|  |  | Froude | 25.226 | 2.343 | 10.764 | 0.000 | 20.542 | 29.910 |  |  |  |  |
|  |  | ClawBefore | 1.972 | 0.854 | 2.309 | 0.024 | 0.265 | 3.678 |  |  |  |  |
|  | hind | Intercept | 46.977 | 4.689 | 10.019 | 0.000 | 34.648 | 59.305 | 0.050 | 0.911 | 80.848 | 8.383 |
|  |  | Froude | 10.117 | 1.957 | 5.171 | 0.000 | 6.206 | 14.027 |  |  |  |  |
|  |  | ClawBefore | 0.127 | 0.713 | 0.179 | 0.859 | -1.297 | 1.552 |  |  |  |  |
| paw length | front | Intercept | 94.652 | 5.757 | 16.442 | 0.000 | 80.352 | 108.95 | 0.263 | 0.851 | 109.211 | 27.697 |
|  |  | Froude | 29.247 | 3.549 | 8.240 | 0.000 | 22.154 | 36.340 |  |  |  |  |
|  |  | ClawBefore | 6.047 | 1.296 | 4.667 | 0.000 | 3.457 | 8.637 |  |  |  |  |
|  | hind | Intercept | 93.353 | 5.213 | 17.909 | 0.000 | 80.752 | 105.96 | 0.095 | 0.765 | 83.858 | 29.487 |
|  |  | Froude | 14.904 | 3.657 | 4.075 | 0.000 | 7.595 | 22.212 |  |  |  |  |
|  |  | ClawBefore | 2.575 | 1.337 | 1.926 | 0.059 | -0.097 | 5.247 |  |  |  |  |
| paw width | front | Intercept | 75.858 | 4.129 | 18.374 | 0.000 | 65.338 | 86.378 | 0.017 | 0.851 | 59.228 | 10.603 |
|  |  | Froude | 4.907 | 2.198 | 2.233 | 0.029 | 0.514 | 9.301 |  |  |  |  |
|  |  | ClawBefore | -0.509 | 0.802 | -0.635 | 0.528 | -2.111 | 1.094 |  |  |  |  |
|  | hind | Intercept | 62.339 | 2.699 | 23.094 | 0.000 | 55.574 | 69.104 | 0.062 | 0.826 | 24.438 | 5.581 |
|  |  | Froude | 6.272 | 1.594 | 3.936 | 0.000 | 3.088 | 9.457 |  |  |  |  |
|  |  | ClawBefore | -0.764 | 0.582 | -1.313 | 0.194 | -1.926 | 0.399 |  |  |  |  |
| relative COP path length | front | Intercept | 7.535 | 0.414 | 18.210 | 0.000 | 6.548 | 8.522 | 0.144 | 0.745 | 0.505 | 0.214 |
|  |  | Froude | -1.615 | 0.311 | -5.187 | 0.000 | -2.238 | -0.993 |  |  |  |  |
|  |  | ClawBefore | 0.016 | 0.114 | 0.139 | 0.890 | -0.212 | 0.244 |  |  |  |  |
|  | hind | Intercept | 7.179 | 0.530 | 13.544 | 0.000 | 5.812 | 8.546 | 0.195 | 0.895 | 0.997 | 0.150 |
|  |  | Froude | -2.432 | 0.262 | -9.287 | 0.000 | -2.955 | -1.908 |  |  |  |  |
|  |  | ClawBefore | -0.136 | 0.095 | -1.428 | 0.158 | -0.327 | 0.055 |  |  |  |  |
| relative cranio-caudal COP Path | front | Intercept | 32.776 | 1.835 | 17.862 | 0.000 | 29.113 | 36.440 | 0.020 | 0.020 | 0.000 | 19.052 |
|  |  | Froude | -0.899 | 2.518 | -0.357 | 0.722 | -5.927 | 4.129 |  |  |  |  |
|  |  | ClawBefore | -1.186 | 1.075 | -1.104 | 0.274 | -3.332 | 0.959 |  |  |  |  |
|  | hind | Intercept | 33.344 | 4.026 | 8.282 | 0.001 | 22.786 | 43.902 | 0.010 | 0.902 | 59.344 | 6.504 |
|  |  | Froude | -2.852 | 1.723 | -1.655 | 0.103 | -6.297 | 0.592 |  |  |  |  |
|  |  | ClawBefore | -1.064 | 0.628 | -1.695 | 0.095 | -2.319 | 0.191 |  |  |  |  |
| relative medio-laterl COP Path | front | Intercept | 20.120 | 2.545 | 7.905 | 0.001 | 13.586 | 26.653 | 0.111 | 0.878 | 22.839 | 3.638 |
|  |  | Froude | -8.128 | 1.288 | -6.311 | 0.000 | -10.702 | -5.554 |  |  |  |  |
|  |  | ClawBefore | -0.990 | 0.470 | -2.108 | 0.039 | -1.928 | -0.051 |  |  |  |  |
|  | hind | Intercept | 10.210 | 1.144 | 8.922 | 0.000 | 7.501 | 12.920 | 0.001 | 0.698 | 3.839 | 1.664 |
|  |  | Froude | 0.073 | 0.868 | 0.084 | 0.934 | -1.662 | 1.807 |  |  |  |  |
|  |  | ClawBefore | -0.147 | 0.318 | -0.462 | 0.646 | -0.781 | 0.488 |  |  |  |  |
| COP speed | front | Intercept | 3.365 | 0.839 | 4.011 | 0.002 | 1.496 | 5.234 | 0.882 | 0.942 | 1.571 | 1.492 |
|  |  | Froude | 22.191 | 0.816 | 27.180 | 0.000 | 20.560 | 23.822 |  |  |  |  |
|  |  | ClawBefore | 0.473 | 0.301 | 1.572 | 0.121 | -0.129 | 1.074 |  |  |  |  |
|  | hind | Intercept | 2.658 | 1.080 | 2.462 | 0.040 | 0.163 | 5.153 | 0.819 | 0.933 | 3.125 | 1.833 |
|  |  | Froude | 22.031 | 0.909 | 24.236 | 0.000 | 20.215 | 23.847 |  |  |  |  |
|  |  | ClawBefore | -0.233 | 0.333 | -0.699 | 0.487 | -0.899 | 0.433 |  |  |  |  |
| Weight distribution | front/ hind | Intercept | 1.413 | 0.074 | 19.162 | 0.000 | 1.242 | 1.583 | 0.021 | 0.648 | 0.015 | 0.008 |
|  |  | Froude | 0.091 | 0.061 | 1.487 | 0.142 | -0.031 | 0.213 |  |  |  |  |
|  |  | ClawBefore | 0.021 | 0.022 | 0.937 | 0.352 | -0.024 | 0.066 |  |  |  |  |
